# Existence and significance of anti-HLA-C autoantibodies to primary and persistent platelet transfusion refractoriness in patients with hematologic disorders: a retrospective study from a single centre

**DOI:** 10.1080/07853890.2024.2446689

**Published:** 2024-12-28

**Authors:** Xunhua Li, Qi Liu, Jingjie Dong, Yaonan Hong, Chuanao Xin, Junfeng Guo, Shan Liu, Peicheng Wang, Zexing Sun, Yingying Shen, Xiawan Yang, Hangchao Li, Yiping Shen, Jianping Shen, Baodong Ye, Yuhong Zhou, Tonglin Hu, Dijiong Wu

**Affiliations:** a Department of Nursing, Chuzhou City Vocational College, Chuzhou Anhui, China; b Department of Hematology, The First Affiliated Hospital of Zhejiang Chinese Medical University (Zhejiang Provincial Hospital of Chinese Medicine), Hangzhou, China; c The First School of Clinical Medicine, Zhejiang Chinese Medical University, Hangzhou, China; d Blood Center of Zhejiang Province, Hangzhou, China; e Department of Clinical Evaluation Center, The First Affiliated Hospital of Zhejiang Chinese Medical University (Zhejiang Provincial Hospital of Chinese Medicine), Hangzhou, China; f National Traditional Chinese Medicine Clinical Research Base (Hematology), Hangzhou, China; g Department of Oncology and Hematology, Wenzhou Hospital of Integrated Traditional Chinese and Western Medicine Affiliated to Zhejiang, Chinese Medicine University, Wenzhou, China

**Keywords:** Platelet transfusion refractoriness, anti-HLA antibody, anti-HLA-C autoantibody, hematologic disorders

## Abstract

**Objectives:**

Platelet transfusion refractoriness (PTR) is a frustrating clinical problem, and primary and persistent (P/P) PTR who experienced persistent PTR since the first transfusion was failed to be well recognized. This study aims to investigate the incidence and risk factors for P/P PTR.

**Methods:**

Patients with hematologic disorders who underwent HLA high-resolution genotyping and donor-specific HLA antibody or panel reactive antibody (PRA) testing between January 2019 and March 2023 were reviewed. Clinical data including infection history, splenomegaly, frequency and quantity of blood transfusions, and transfusions response were delineated and subsequently analyzed.

**Results:**

114 patients were included retrospectively, and 1071 transfusions were recorded. The overall incidence of PTR was 28.95% (33/114), with 63.63% (21/33) being P/P PTR. Anti class I HLA (anti-HLA-I) antibody was identified as an independent risk factor for ineffective platelet transfusion through multivariate logistic regression analysis (*p* = .034). Interestingly, anti-HLA-C autoantibodies were first found in six patients, and both anti-HLA-A and C autoantibodies were detected in one case, comprising a total of 10.71% (6/56) of HLA-I antibody-positive patients. Further analysis revealed that anti-HLA-C autoantibody was identified as an independent risk factor for P/P PTR (*p* = .039). Among patients with positive anti-HLA-C antibodies, significant differences in the effectiveness of ABO, D-matched and cross-matching transfusions were observed between patients with or without anti-HLA-C autoantibodies (*p* < .001 and *p* = .017). Notably, platelet transfusions independence was achieved by two of the four patients who received rituximab.

**Conclusions:**

This work emphasized the significance of anti-HLA-C autoantibody for P/P PTR in hematological patients, and rituximab may therapeutic.

## KEY MESSAGES

- 28.95% who experienced platelet transfusion refractoriness (PTR), 23 were primary and persistent (P/P) PTR
- Anti-HLA-C autoantibody was identified as an independent risk factor for P/P PTR
- Application of rituximab may practical in the management of P/P PTR with HLA-C autoantibodies

28.95% who experienced platelet transfusion refractoriness (PTR), 23 were primary and persistent (P/P) PTR

## Introduction

Platelet transfusion plays a crucial role in the supportive treatment of hematologic disorders, particularly in conditions like aplastic anemia (AA), myelodysplastic syndrome (MDS), and post-chemotherapy myelosuppression [1,2]. However, in patients who have undergone multiple transfusions or suffer from immune-mediated diseases, platelet transfusion refractoriness (PTR) can occasionally occur. PTR can be defined as the failure to achieve a satisfactory post-transfusion increase in platelet count, leading to an elevated risk of bleeding, increased hospitalization costs, and reduced survival rates [3–6]. To date, numerous factors contributing to PTR have been explored, encompassing both immune and non-immune factors. Immune factors include the formation of antibodies against platelet antigens, resulting in alloimmunization, which can be categorized into two groups: human leukocyte antigen (HLA) and human platelet antigen (HPA) systems. Alloimmunization induced by HLA antigens is more prevalent than that by the HPA system and is considered the primary cause of immune-mediated PTR [7]. Non-immune factors leading to refractoriness are mainly associated with patients’ clinical conditions and related treatment regimens, such as infections, sepsis, splenomegaly, body temperature, and the administration of cytotoxic drugs and antibiotics [8,9].

In recent times, an increasing number of studies have focused on understanding the causes and finding solutions for PTR [8,10]. It has been reported that patients undergoing liver transplantation who test positive for anti class I HLA (anti-HLA-I) antibodies are more susceptible to PTR [11], as are patients undergoing hematopoietic stem cell transplantation (HSCT) [12]. Based on our clinical experience, we have also observed cases of primary and persistent (P/P) PTR, where patients experience persistent PTR from their first platelet transfusion. These patients suffer from severe thrombocytopenia, which can only be partially corrected with cross-matched platelet transfusions. However, existing studies have paid limited attention to these specific group of patients (P/P PTR). Therefore, we conducted a retrospective study to determine the occurrence rate of P/P PTR and analyze the risk factors in patients with hematologic disorders, with a particular focus on HLA antibodies.

## Methods

### Study design

To explore the factors contributing to the PTR, especially P/P PTR, we focus on the significance of HLA antibodies expression, especially autoantibodies. Patients who have the HLA high-resolution genotyping and HLA antibody test reports, and experienced platelets transfusion were reviewed between January 2019 and March 2023 in our Hospital, the First Affiliated Hospital of Zhejiang Chinese Medical University. Clinical data, including age, gender, type of hematologic disorders, expression of HLA antibodies, presence of infection (fever and administration of antibiotics), splenomegaly, frequency and quantity of blood transfusions, C-reactive protein (CRP) levels were collected and analyzed.

### Ethics approval and consent to participate

This study was approved by the ethical committee of the First Affiliated Hospital of Zhejiang Chinese Medical University (NO. 2023-KL-287-01), and was registered at *chictr.org.cn* as #ChiCTR2300077945. All procedures performed in studies involving human participants were in accordance with the ethical standards of the institutional and/or national research committee and with the 1964 Helsinki Declaration and its later amendments or comparable ethical standards. Written informed consent was obtained from all the participants.

### Patient enrollment

Inclusion criteria for patients were as follows: (1) confirmed diagnosis of hematologic disorders; (2) availability of HLA high-resolution genotyping test reports (indicating potential suitability for hematopoietic stem cell transplantation) and either a donor-specific HLA antibodies (DSA) test or panel reactive antibodies (PRA) testing report; (3) had received a minimum of two platelet transfusions; (4) no previous history of transfusions before admission. Exclusion criteria included a lack of information regarding baseline characteristics.

### Platelets source and transfusion management

All apheresis platelets were supplied by the Zhejiang Provincial Blood Center. The preparation and storage of apheresis platelets complied with the quality standards for whole blood and blood components (GB18469-2012) [13]. Prior to transfusion, patients underwent ABO blood group determination, Rh blood group typing, and screening for irregular antibodies. All blood products were administered according to the same ABO and Rh (D) blood type.

### Definitions and evaluation

The response to platelet transfusions was assessed using the corrected count increment (CCI). CCI is determined based on the post-transfusion platelet increment (PPI), the quantity of platelets transfused, and the patient’s body surface area, which is calculated using the Stevenson formula. PTR was defined as a corrected count increment of CCI less than 5000 at 24h after two sequential platelet transfusion [14]. P/P PTR was defined as: (1) meeting diagnostic criteria for PTR; (2) primary PTR: experienced CCI less than 5000 at 24h since the first platelet transfusion; (3) persistent PTR: overall effective platelet transfusion was calculated less than 30% accompanying with no consecutive effective transfusion. The relevant formulas are as follows:

$$\begin{array}{c} \text{PPI}=\text{post}-\;\text{transfusion}\;\text{platelet}\;\text{count}\\ -\text{pretransfusion}\;\text{platelet}\;\text{count} \end{array}$$

$$\begin{array}{c} \text{Body}\;\text{surface}\;\text{area}(m^{2})=0.0061\times \text{height}(\text{cm})\\ +0.0128\times \text{weight}(\text{kg})-0.1529 \end{array}$$

CCI=PPI(/I)×bodysurface area(m2)Numberof platelet transfused(1011)

### HLA high-resolution genotyping test and HLA antibodies test

High-resolution HLA genotyping was conducted through the Polymerase Chain Reaction- Sequence Specific Primer (PCR-SSP) sequencing method. Meanwhile, HLA antibodies were detected using HLA specific antibody detection kit (LABScreen^®^, ONE LAMBDA), which utilize purified HLA antigen-coated microbeads to detect HLA antibodies by flow cytometry, and it defined mean fluorescent intensity (MFI) >500 as positive for an antibody [15]. Specifically, For LABScreen PRA or LABScreen Single Antigen, the normalized fluorescent value for each HLA coated bead equals the value of that bead divided by the value of the negative control bead (NC bead). For LABScreen Mixed, the normalized fluorescent signal equals the value of the Class I or Class II coated bead minus the value of the NC bead. Both these examinations were conducted at the Zhejiang Provincial Blood Center. As noted, if a DSA or PRA test reported the antibody which was matched the allele tested by high-resolution genotyping, it is diagnosed as HLA autoantibodies.

## Statistical analysis

Data collection and analysis were carried out using Excel and SPSS version 27.0. Descriptive reporting was employed to summarize the baseline characteristics of the patients. Comparisons between the anti-HLA-C autoantibody-positive and -negative groups were conducted using either the Mann–Whitney *U*-test or the test, as appropriate. The cutoff value for CRP was determined through receiver operating characteristic (ROC) curve analysis. The median CRP value was established as the cutoff value, based on the ROC curve’s lack of discriminatory ability to predict PTR. Binary logistic regression was utilized to analyze the factors influencing PTR and P/P PTR. A significance level of *p* < .05 was considered statistically significant.

## Results

### Patient characteristics

A total of 114 patients were included in the study, with a median age of 40 years (range: 14–80 years). Among them, 46 patients were male (40.35%), and the distribution of hematologic disorders in this cohort was as follows: AA (65.79%), leukemia (21.93%), MDS (8.77%), and other hematologic conditions (3.51%). Out of the 68 female patients, 16 (23.53%) had never been pregnant. Before receiving platelet transfusions, 76 out of 114 patients (66.67%) had experienced fever, while 57 out of 114 patients (50%) exhibited elevated CRP levels (Table 1).

**Table 1.** Patients’ baseline characteristics.

Characteristic	Full population (*n* = 114)
Gender (male/female)	46/68
Age (years): median (range)	40 (14-80)
Disease distribution	
Aplastic anemia, *N* (%)	75 (65.79%)
Leukaemia, *N* (%)	25 (21.93%)
Myelodysplasia syndrome, *N* (%)	10 (8.77%)
Other, *N* (%)	4 (3.51%)
Fever	
Yes, *N* (%)	76 (66.67%)
No, *N* (%)	38 (33.33%)
C-reactive protein	
<6.23 mg/L, N (%)	57 (50%)
≥6.23 mg/L, N (%)	57 (50%)
Splenomegaly	
Yes, *N* (%)	16 (14.04%)
No, *N* (%)	98 (85.96%)
Previous administration of antibiotics	
Yes, *N* (%)	108 (94.74%)
No, *N* (%)	6 (5.26%)
Numbers of pregnancy	
0, *N* (%)	16 (23.53%)
1, *N* (%)	29 (42.65%)
2, *N* (%)	20 (29.41%)
3, *N* (%)	3 (4.41%)

### Platelet transfusion response

In this cohort, the incidence of PTR was 28.95% (33/114), with 63.63% (21/33) classified as P/P PTR. A total of 1071 ABO, D-matched platelet transfusions were administered to the 114 patients during the follow-up period, of which 770 were deemed effective transfusions. Out of 69 cross-matching platelet transfusions, 39 achieved a satisfactory response. Besides, effective platelet transfusions were also calculated in 33 PTR and 21 P/P PTR patients. A total of 330 and 193 ABO-D matched platelet transfusions were given to 33 PTR and 21 P/P PTR patients in the cohort, of which 122 (36.97%) and 44 (22.80%) were deemed effective transfusions, respectively. As for cross-matching platelet transfusions, the effective response rate was 46.94% (23/49) and 44.44% (12/27) in 33 PTR and 21 P/P PTR patients. Of course, there were 80% (16/20) effective cross-matching platelet transfusions in a total number of 81 patients without PTR. Of noted, 11 patients with PTR conducted an HLA high-resolution genotyping test in an effort for gene-matching platelet transfusions. Eventually, 6 patients received gene-matching platelet transfusions, and 5 of whom achieved platelet increments.

### Univariate logistic regression analysis for PTR and P/P PTR

Firstly, univariate logistic regression analysis was conducted to assess factors influencing platelet transfusion efficacy, including PTR and P/P PTR. The potential factors considered in the analysis included gender, age, fever, CRP subgroups (<6.23 and ≥6.23), splenomegaly, administration of antibiotics, presence of anti-HLA-I antibodies, number of pregnancies, and the type of hematologic disease. The results showed that anti-HLA-I antibodies were both an independent risk factor for PTR ((odds ratio [OR] = 2.765, 95% confidence interval [CI]: 1.184–6.453, *p* = .019) and P/P PTR (OR = 3.171, 95%CI: 1.130–8.894, *p* = .028)), while CRP subgroups (OR = 3.036, 95%CI: 1.083–8.512, *p* = .035), splenomegaly (OR = 4.667, 95%CI: 1.495–14.567, *p* = .008) and leukemia (OR = 3.059, 95%CI: 1.047–8.936, *p* = .041) were only for P/P PTR (Table 2).

**Table 2.** Univariate logistic regression analysis for PTR and P/P PTR.

	PTR			P/P PTR		
	OR	95%CI	*p* value	OR	95%CI	*p* value
Gender	1.522	0.652–3.549	.331	1.444	0.533–3.912	*.469*
Age	0997	0.969–1.025	.827	1.012	0.979–1.045	*.483*
Fever	1.215	0.508–2.907	.662	1.000	0.366–2.713	*1.000*
CRP subgroups	2.187	0.950–5.035	.066	3.036	1.083–8.512	***.035***
Splenomegaly	2.920	0.992–8.599	.052	4.667	1.495–14.567	***.008***
Administration of antibiotics	2.105	0.236–18.743	.505	1.136	0.126–10.268	*.909*
Anti-HLA-I antibodies	2.765	1.184–6.453	.019	3.171	1.130–8.894	***.028***
Disease (aplastic anemia)^a^	Reference		.578	Reference		*.234*
Disease (leukaemia)	1.387	0.516–3.727	.517	3.059	1.047–8.936	***.041***
Disease (myelodysplasia syndrome)	1.965	0.500–7.177	.333	1.625	0.301–8.776	*.573*
Disease (others)	2.947	0.388–22.394	.296	2.167	0.205–22.926	*.521*

PTR: platelet transfusions refractoriness; OR: odds ratio; CI: confidence interval; CRP: C-reactive protein.

^a^ Aplastic anemia as reference.

### Anti-HLA antibodies distribution

To better understanding the value of anti-HLA-I antibodies, anti-HLA antibodies were categorized into two groups: anti-HLA-I and anti-HLA-II antibodies. Based on the results of the HLA high-resolution genotyping test and HLA antibodies test, 56 out of 114 patients (49.12%) tested positive for anti-HLA-I antibodies, while 42 out of 114 patients (36.84%) tested positive for anti-HLA-II antibodies. Within the anti-HLA-I antibodies group, patients were further divided into three subgroups based on gene loci: anti-HLA-A, B, and C antibodies. The distribution was as follows: 30.7% positive for anti-HLA-A, 42.11% positive for anti-HLA-B, and 28.07% positive for anti-HLA-C antibodies. There were 9, 0 and 7 patients simultaneously presenting with anti-HLA-A and B antibodies, anti-HLA-A and C antibodies, anti-HLA-B and C antibodies, respectively. As noted, 23 out of 114 patients (20.18%) tested concurrently positive for anti-HLA-A, B and C antibodies. Interestingly, it was observed that 6 patients had anti-HLA-I autoantibodies, including 6 with antibodies against HLA-C, and 1 patient had both HLA-A and C autoantibodies (Table 3).

**Table 3.** Anti-HLA Antibodies distribution.

Anti-HLA antibodies	*N* (%)
Anti-HLA-I antibodies	
Positive	56 (49.12%)
Negative	58 (50.88%)
HLA-A	
Positive	35 (30.70%)
Negative	79 (69.30%)
HLA-B	
Positive	48 (42.11%)
Negative	66 (57.89%)
HLA-C	
Positive	32 (28.07%)^a^
Negative	82 (71.93%)
Anti-HLA-A autoantibodies	
Positive	1 (0.88%)
Negative	113 (99.12%)
Anti-HLA-B autoantibodies	
Positive	0 (0%)
Negative	114 (100%)
Anti-HLA-C autoantibodies	
Positive	6 (5.26%)
Negative	108 (94.76%)
Anti-HLA-II antibodies	
Positive	42 (36.84%)
Negative	72 (63.16%)

* Two patients are only positive with anti-HLA-C antibodies (lacking anti-HLA-A and B antibodies).

Comparisons based on PTR in all patients or P/P PTR in PTR patients were conducted. The results showed that there were significant differences in the distribution of anti-HLA-A, B, C antibodies and anti-HLA-C autoantibodies between PTR and non-PTR patients (Supplemental Table 1). However, no differences were observed based on P/P PTR in patients with PTR (Supplemental Table 2).

### The platelet transfusion response based on locus of HLA-I subgroups

**Figure 1.**
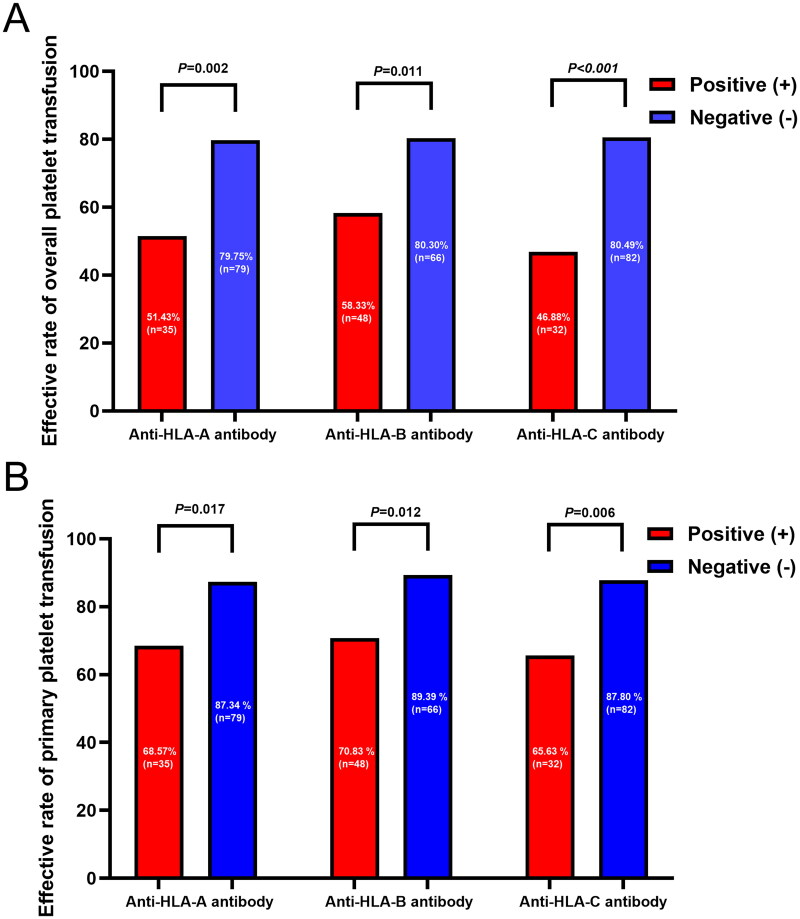
The platelet transfusion response based on locus of HLA-I subgroups. The occurrence rate of overall (A) and primary (B) platelet transfusion efficacy was compared based on locus of HLA-I subgroups, and found that either anti-HLA-A, HLA-B or HLA-C antibody positive contribute to the ineffective to the platelet transfusion (all *p* < .05).

Among patients positive for anti-HLA-A antibodies, 51.43% (18/35) and 68.57% (24/35) achieved an overall and primary platelet transfusion response, respectively, while 79.75% (63/79) and 87.34% (69/79) of patients negative for anti-HLA-A antibodies exhibited a positive response. In patients positive for anti-HLA-B antibodies, the occurrence rate of overall and primary platelet transfusion efficacy was 58.33% (28/48) and 70.83% (34/48), respectively, compared to 80.30% (53/66) and 89.39% (59/66) in patients negative for anti-HLA-B antibodies. For patients with anti-HLA-C antibodies, 46.88% (15/32) and 65.63% (21/32) achieved an overall and primary platelet transfusion response, while 80.49% (66/82) and 87.80% (72/82) of patients without anti-HLA-C antibodies exhibited a positive response. Statistical analysis indicated that the presence of anti-HLA-A, HLA-B, or HLA-C antibodies contributed to ineffective overall and primary platelet transfusion responses (all *p* < .05) (Figure 1A,B).

### Logistic regression identified anti-HLA-C autoantibodies as the risk factor for P/P PTR

**Figure 2.**
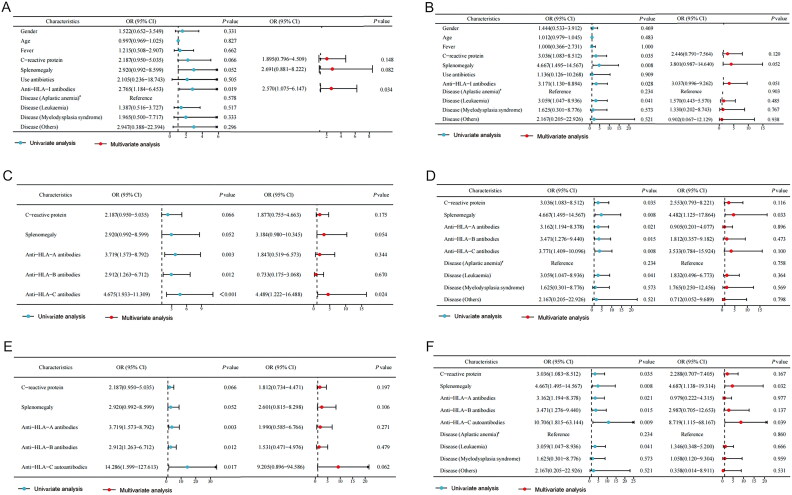
Multivariate logistic regression identify anti-HLA-C autoantibodies as the risk factor for primary and persistent (P/P) platelet transfusion refractoriness. Factors with a univariate logistic regression result of *p* < .1 were included in the multivariate logistic regression analysis. The multivariate logistic regression analysis revealed that anti-HLA-I antibodies were an independent risk factor for PTR (2 A), but not in P/P PTR (2B); anti-HLA-C antibody was an independent risk factor for overall PTR (2 C), but not for P/P PTR (2D). Considering that anti-HLA-C antibody and anti-HLA-C autoantibody can be confounding factors, only anti-HLA-C autoantibody was selected for logistic regression analysis alongside anti-HLA-a and B antibodies. Result showed that no factors could predict the occurrence of PTR (2E), while anti-HLA-C autoantibody positive was the independent risk factor for P/P PTR (2 F). PTR: platelet transfusion refractoriness.

Factors with a univariate logistic regression result of *p* < .1 were included in the multivariate logistic regression analysis. The multivariate logistic regression analysis revealed that anti-HLA-I antibodies were an independent risk factor for PTR (OR = 2.570, 95%CI: 1.075–6.147, *p* = .034) (Figure 2A), but not in P/P PTR (Figure 2B).

To further elucidate the significance of anti-HLA-I antibodies in both PTR and P/P PTR, we included each locus of HLA-I in the logistic regression analysis. The results indicated that anti-HLA-C antibody was an independent risk factor for PTR (Figure 2C), but not for P/P PTR (Figure 2D). Considering that anti-HLA-C antibody and anti-HLA-C autoantibody can be confounding factors, only anti-HLA-C autoantibody was selected for logistic regression analysis alongside anti-HLA-A and B antibodies. The findings demonstrated that only anti-HLA-C autoantibody positive could nearly predict the occurrence of PTR (OR = 9.205, 95%CI: 0.896–94.586, *p* = .062, Figure 2E), while splenomegaly and anti-HLA-C autoantibody positive were both the independent risk factor for P/P PTR (Figure 2F).

### Patients with anti-HLA-C autoantibodies experienced more ineffective ABO, D-matched platelet transfusions than those without in anti-HLA-C antibody positive population

**Figure 3.**
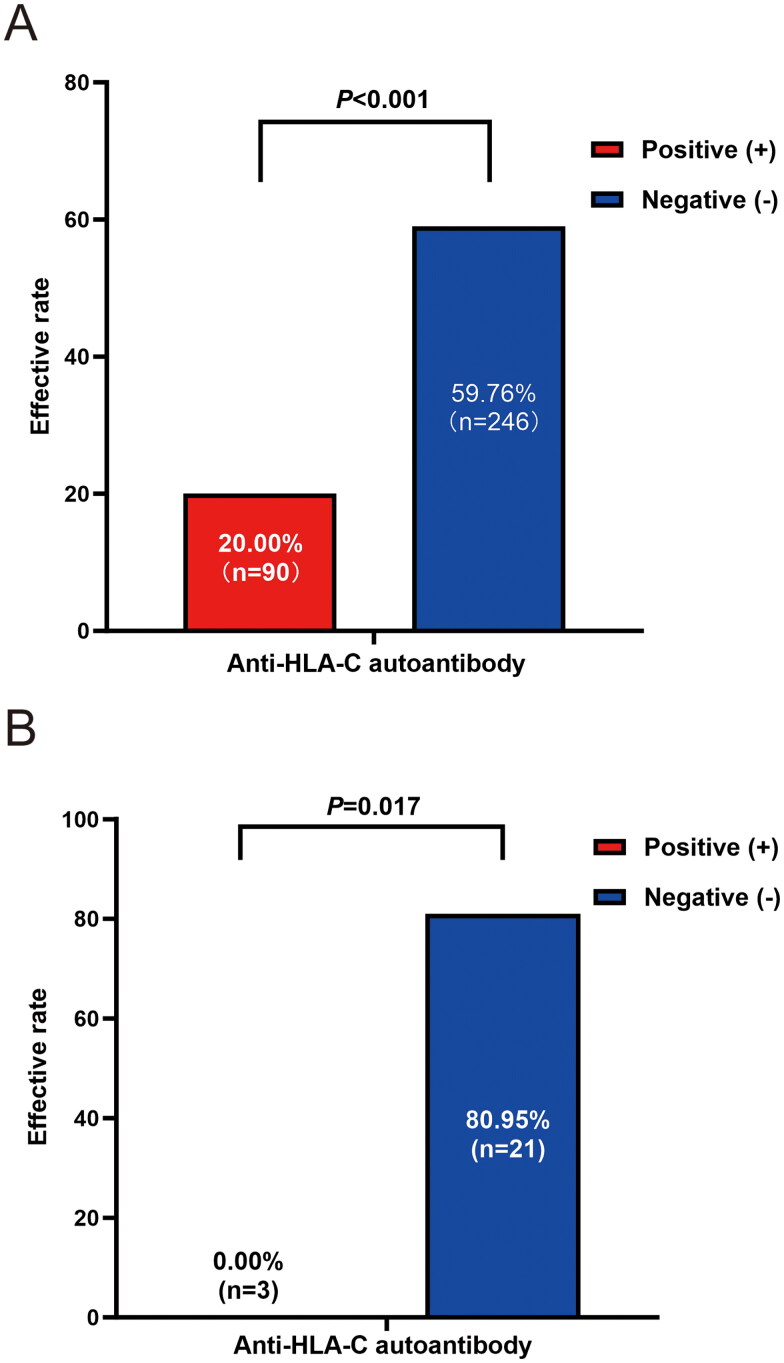
Comparison Of the ABO-D matched and cross-matching platelet transfusions efficiency in patients with or without anti-HLA-C autoantibodies in anti-HLA-C antibody positive population. Significant differences were observed in both effective ordinary (*p* < .001, 3 A) and cross-matching (*p* = .017, 3B) platelet transfusion between patients with or without anti-HLA-C autoantibodies (*n* = 6 *vs. n* = 26).

The baseline characteristics of patients who tested positive for anti-HLA-C antibodies, categorized based on the presence or absence of anti-HLA-C autoantibodies, are summarized in Table 4. Age, gender, disease distribution, fever occurrence, white blood cell count (WBC), splenomegaly, and the administration of antibiotics were similar between patients positive and negative for anti-HLA-C autoantibodies. Remarkably, we observed that the effectiveness of overall platelet transfusions in the 6 patients with anti-HLA-C autoantibodies was only 20% (18 out of 90 transfusions), while for patients lacking anti-HLA-C autoantibodies, it was significantly higher at 59.76% (147 out of 246 transfusions) (*p* < .001) (Figure 3A).

**Table 4.** Comparison of baseline characteristics in patients with or without anti-HLA-C autoantibodies in anti-HLA-C antibody positive population.

Characteristics	Anti-HLA-C autoantibodies		
	Positive (*n* = 6)	Negative (*n* = 26)	*p* value
Gender			
Male, *N* (%)	2	7	1.000
Female, *N* (%)	4	19	
Age (years):			.410
≤40, N (%)	1 (16.7%)	17 (65.4%)	
>40, N (%)	5 (83.3%)	9 (34.6%)	
Disease distribution			.298
Aplastic anemia, *N* (%)	2 (33.3%)	19 (73.1%)	
Leukaemia, *N* (%)	2 (33.3%)	4 (15.4%)	
Myelodysplasia syndrome, *N* (%)	1 (16.7%)	1 (3.8%)	
Other, *N* (%)	1 (16.7%)	2 (7.7%)	
Fever			.647
Yes, *N* (%)	3 (50%)	17 (65.4%)	
No, *N* (%)	3 (50%)	9 (34.6%)	
C-reactive protein			.672
≥6.23mg/L, *N* (%)	4 (66.7%)	14 (53.8%)	
<6.23mg/L, *N* (%)	2 (33.3%)	12 (46.2%)	
Splenomegaly			1.000
Yes, *N* (%)	1 (16.7%)	3 (11.5%)	
No, *N* (%)	5 (83.3%)	23 (88.5%)	
Previous administration of antibiotics			.345
Yes, *N* (%)	5 (83.3%)	25 (96.2%)	
No, *N* (%)	1 (16.7%)	1 (3.8%)	
PTR			.178
Yes, *N* (%)	5 (83.3%)	12 (46.2%)	
No, *N* (%)	1 (16.7%)	14 (53.8%)	
P/P PTR			.148
Yes, *N* (%)	4 (66.7%)	7 (26.9%)	
No, *N* (%)	2 (33.3%)	19 (73.1%)	

### Patients with anti-HLA-C autoantibodies failed to respond to cross-matching platelet transfusions

Among the six patients with positive anti-HLA-C autoantibodies, three received cross-matching platelet transfusions, and all of them experienced ineffective transfusions. In contrast, among the patients with anti-HLA-C antibodies but not autoantibodies, 21 received cross-matching platelet transfusions, and 17 of them responded favorably, demonstrating a significant difference (*p* = .017) (Figure 3B).

### Patients with positive anti-HLA-C autoantibodies tend to coexist with anti-HLA-A, B, and C antibodies

In our study, 5 out of 6 patients with anti-HLA-C autoantibodies were simultaneously positive for anti-HLA-A, B and C antibodies. 83.3% (5/6) and 16.7% (18/108) of patients were concurrently positive for anti-HLA-A, B and C antibodies in presence or absence of anti-HLA-C autoantibodies. A significant difference was observed (χ^2^=15.687, *p* = .001).

### Outcomes of patients positive for anti-HLA-C autoantibodies

Among the six patients who tested positive for anti-HLA-C autoantibodies, only one AA patient response to platelet transfusion (cured after allogeneic hematopoietic stem cell transplantation, allo-HSCT), all the other five patients experienced PTR (5/5) or P/P PTR (4/5) (detail in Table 5 and Supplemental Table 3). To correct the PTR, the application of rituximab (RTX), intravenous immunogloblin (IVIG), plasmapheresis, gene-matching platelet transfusions, as well as salvage HSCT were performed in selected cases. Two patients (2/4) response to RTX and got hematological improvement; two patients received salvage allo-HSCT, both died ascribe to multiple organ failure and intracranial bleeding before engraftment; one AA received anti-thymocyte globulin (ATG) treatment but unfortunately succumbed to severe gastrointestinal bleeding before hematopoietic recovery.

**Table 5.** Details of patients with positive anti-HLA-C autoantibodies.

	PT1	PT2	PT3	PT4	PT5	PT6
Disease	AA	Pancytopenia	AML	AML	MDS	AA
Gender	F	F	F	F	M	M
Age (years)	63	33	52	42	44	45
Number of pregnancies	2	2	1	1	NA	NA
Fever	NO	NO	YES	YES	YES	NO
CRP (mg/L)	23.85	1.5	96.41	8	7.55	1.92
Splenomegaly	NO	YES	NO	NO	NO	NO
Administration of antibiotics	NO	YES	YES	YES	YES	YES
Anti-HLA-I antibodies	(+)	(+)	(+)	(+)	(+)	(+)
Anti-HLA-A antibodies	(+)	(–)	(+)	(+)	(+)	(–)
Anti-HLA-B antibodies	(+)	(+)	(+)	(+)	(+)	(–)
Anti-HLA-C antibodies	(+)	(+)	(+)	(+)	(+)	(+)
Anti-HLA-II antibodies	(+)	(–)	(+)	(–)	(+)	(–)
Anti-HLA-C autoantibody						
Gene loci	C*03:03	C*15:02	C*07:02	C*14:02	C*15:02	C*07:02
MFI	550.4	1082.82	1239.29	900.69	518.42	723.49
PTR	YES	YES	YES	YES	YES	NO
P/P PTR	YES	YES	YES	NO	YES	NO
Treatment and response						
RTX	NO	YES (R)	YES (NR)^a^	YES(R)	YES (NR)^a^	NO
Plasma exchange	NO	NO	NO	YES(NR)^a^	YES (NR)^a^	NO
IVIG	YES (NR)	NO	YES (NR)	NO	YES (NR)	NO
Gene-matching platelet transfusion	NO	YES (NR)	NO	NO	NO	NO
Outcomes	Died (gastrointestinal hemorrhage)	Survive	Died (multiple organ failure)	Survive	Died (intracranial hemorrhage)	Survive

PT: patient; F: female; M: male; AA: aplastic anemia; AML: acute myeloid leukemia; MDS: myelodysplastic syndrome; CRP: C-reactive protein; MFI: mean fluorescence intensity; PTR: platelet transfusions refractoriness; P/P: primary and persistent; RTX: rituximab; IVIG: intravenous immunogloblin; NA: not applicable; R: response; NR: no response.

^a^ Targeting the donor specific antibody (DSA) prior to conditioning of allogeneic hematopoietic stem cell transplantation (allo-HSCT).

## Discussion

Platelet transfusions are crucial for preventing and treating bleeding in patients with hematologic disorders, especially those with bone marrow failure and post-chemotherapy myelosuppression. However, frequently and repeated platelet transfusions can lead to PTR, characterized by ineffective transfusion responses. Current studies have focused on factors and mechanisms associated with PTR, but there has been limited research attention given to P/P PTR. This study was designed to systematically assess the occurrence of P/P PTR in the context of hematologic disorders and identify potential factors contributing to P/P PTR to optimize treatment approaches.

Our study revealed that the occurrence rate of PTR was 28.95%, which aligns with previous literature reports [8]. Importantly, we provided the first data showing that the occurrence rate of P/P PTR was 18.42%, which occupied 63.63% in the PTR. Logistic analysis was conducted to identify factors contributing to ineffective transfusions, and it was found that anti-HLA-I antibodies emerged as an independent risk factor for PTR. Anti-HLA-I antibodies are a major cause of immune-mediated PTR and are responsible for approximately 80% of immune-mediated PTR cases [10,16]. The mechanisms underlying the pathogenicity of these antibodies in PTR include: (a) the induction of FcγRIIa-dependent platelet activation and enhanced phagocytosis by macrophages [17]; (b) the activation of complement through the deposition of C4b and C3b and the formation of the membrane-attack complex (MAC) [18]. Although platelet surfaces contain multiple blood group antigens, such as ABO, HLA-I, HPA, and CD36 antigens, the high polymorphism of HLA-A and HLA-B antigens makes recipients more likely to produce HLA-I antibodies during platelet transfusion [19]. Therefore, HLA-A and HLA-B have become the primary targets of research on HLA-I antibodies. Gao et al. found that HLA-A24:02 may be a susceptible gene for producing anti-HLA-I antibodies in patients with malignant hematological diseases, while HLA-A30:01 and HLA-B*13:02 act as protective genes, revealing a correlation between the production of anti-HLA-I antibodies and HLA-A and HLA-B genes [20]. Additionally, there have been reports of cases in which anti-HLA-I antibodies played a significant role in platelet clearance. For example, one neonate with marked thrombocytopenia tested positive for anti-HLA-B55 antibodies, but these antibodies disappeared on the 20th day, coinciding with an increase in platelet count [21]. Another case showed the continuous appearance of HLA-I antibodies (anti-HLA-A2, anti-HLA-A24, and anti-HLA-B44) during the duration of significant PTR following peripheral blood progenitor cell transplantation (PBPCT) [22]. These cases highlight the important role of anti-HLA-I antibodies in platelet clearance.

Until recently, patients who did not respond to ABO, D-matched platelet transfusions were routinely screened for alloantibodies against anti-HLA-A, anti-HLA-B, anti-HLA-C, and anti-HLA-DR. However, not all patients underwent HLA autoantibody testing, primarily because HLA high-resolution genotyping tests were typically conducted in populations potentially eligible for HSCT. Consequently, there was a lack of literature exploring the association between autoantibodies and platelet transfusions. In this study, we screened 114 patients and identified only 6 patients with anti-HLA-C autoantibodies, indicating a prevalence of 5.26%. Surprisingly, we found that anti-HLA-C autoantibodies were associated with both PTR and P/P PTR. Saito et al. previously identified six alloimmunized patients who were refractory to HLA-A and HLA-B-compatible platelet transfusions but showed increased platelet transfusion increments when they received HLA-C-compatible transfusions due to the presence of anti-HLA-Cw3, Cw-7, or Cw8 antibodies [23]. Han’s research also revealed that AML patients with specific HLA-C genotypes (C1C1 and C1C2) were unresponsive to platelet transfusions from donors with C2C2 genotypes, highlighting the connection between HLA-C genotype and platelet transfusion efficacy [24]. These findings clearly indicate that HLA-C antibodies play a significant role in the effectiveness of platelet transfusions. However, existing literature does not report on the occurrence of anti-HLA-C autoantibodies leading to ineffective platelet transfusions. In our cohort, we discovered there were 6 patients with positive anti-HLA-C autoantibodies, of whom 5 coexist with anti-HLA-A, B, and C antibodies, implying patients who were simultaneously positive for HLA-A, B and C antibodies seemed to be more immune imbalanced, indirectly leading to produce anti-HLA-C autoantibodies. Hence, we hypothesized that patients with hematologic disorders and anti-HLA-C autoantibodies were in a state of immune overactivation, perceiving infused platelets as foreign objects, and consequently, these platelets were not able to fulfill their intended therapeutic function.

In terms of managing platelet refractoriness caused by anti-HLA-C autoantibodies, traditional approaches to identifying compatible platelet units, such as cross-matching or even gene-matching, have not yielded satisfactory results. Our previous research revealed that patients with PTR showed improvement in platelet transfusion when treated with rituximab. The underlying mechanism involved the opsonization of B cells to block macrophages. However, these PTR patients were not tested for anti-HLA autoantibodies [25,26].

The limitation of this study mainly derived from the attention to effect of independent anti-HLA antibodies on PTR, failed to analyze influence of the combination of anti-HLA antibodies, as well as MFI and PRA, which were demonstrated to be independent factors in immune-mediated refractoriness in literatures [27]. Besides, the study was retrospective with a limited sample size of patients with anti-HLA-C autoantibodies. Furthermore, 5 out of 6 patients with anti-HLA-C autoantibodies were simultaneously positive for anti-HLA-A, B and C antibodies in addition to all complicated with one or more non-immune risk factors. Although the immune and non-immune risk factors were included into logistic regressions for PTR, we cannot determine whether these factors contributed to the formation of anti-HLA-C autoantibodies.

In summary, our data emphasize the importance of detecting anti-HLA-C autoantibodies through HLA high-resolution genotyping tests for patients experiencing ineffective platelet transfusions, especially those with P/P PTR. Rituximab could be considered a potential and universally applicable therapy for PTR.

## Supplementary Material

Supplemental Material

## Data Availability

The data used to support the study are available from the corresponding author upon request.
