# Supplementary material for: Existence and significance of anti-HLA-C autoantibodies to primary and persistent platelet transfusion refractoriness in patients with hematologic disorders: a retrospective study from a single centre
Source: Ann Med. 2024 Dec 28;57(1):2446689. doi: 10.1080/07853890.2024.2446689 (PMC11703460; doi:10.1080/07853890.2024.2446689)
Supplement: Supplemental Material [file IANN_A_2446689_SM6790.zip › Suppl_Mat/Supplemental Table 1 .docx]

**Supplemental Table 1. The distribution of anti-HLA antibodies**

**between PTR and non-PTR patients**

| **Anti-HLA antibodies** | **PTR (n=33)** | **Non-PTR (n=81)** | ***P* value** |
| --- | --- | --- | --- |
| Anti-HLA-I antibodies  Positive  Negative  HLA-A  Positive  Negative  HLA-B  Positive  Negative  HLA-C  Positive  Negative  Anti-HLA-A autoantibodies  Positive  Negative  Anti-HLA-B autoantibodies  Positive  Negative  Anti-HLA-C autoantibodies  Positive  Negative | 22 (66.67%)  11 (33.33%)  17 (51.52%)  16 (48.48%)  20 (60.61%)  13 (39.39%)  17 (51.52%)  16 (48.48%)  1 (3.03%)  32 (96.97%)  0 (0%)  33 (100%)  5 (15.15%)  28 (84.85%) | 34 (41.98%)  47 (58.02%)  18 (22.22%)  63 (77.78%)  28(34.57%)  53 (65.43%)  15 (18.52%)  66 (81.48%)  0 (0%)  81 (100%)  0 (0%)  81 (100%)  1 (1.23%)  80 (98.77%) | 0.017  0.002  0.011  0.001  0.289  ---  0.008 |
