# Supplementary material for: Existence and significance of anti-HLA-C autoantibodies to primary and persistent platelet transfusion refractoriness in patients with hematologic disorders: a retrospective study from a single centre
Source: Ann Med. 2024 Dec 28;57(1):2446689. doi: 10.1080/07853890.2024.2446689 (PMC11703460; doi:10.1080/07853890.2024.2446689)
Supplement: Supplemental Material [file IANN_A_2446689_SM6790.zip › Suppl_Mat/Supplemental Table 2 .docx]

**Supplemental Table 2. The distribution of anti-HLA antibodies in patients with PTR**

| **Anti-HLA antibodies** | **P/P PTR**  **Yes (n=21) No (n=12)** | | | ***P* value** |
| --- | --- | --- | --- | --- |
| Anti-HLA-I antibodies  Positive  Negative  HLA-A  Positive  Negative  HLA-B  Positive  Negative  HLA-C  Positive  Negative  Anti-HLA-A autoantibodies  Positive  Negative  Anti-HLA-B autoantibodies  Positive  Negative  Anti-HLA-C autoantibodies  Positive  Negative | | 15 (71.43%)  6 (28.57%)  11 (52.38%)  10 (47.62%)  14 (66.67%)  7 (33.33%)  11 (52.38%)  10 (47.62%)  0 (0%)  21 (100%)  0 (0%)  21 (100%)  4 (19.05%)  17 (80.95%) | 7 (58.33%)  5 (41.67%)  6 (50.00%)  6 (50.00%)  6 (50.00%)  6 (50.00%)  6 (50.00%)  6 (50.00%)  1 (8.33%)  11 (91.67%)  0 (0%)  12 (100%)  1 (8.33%)  11 (91.67%) | 0.471  1.000  0.465  1.000  0.364  ---  0.630 |
