# Supplementary material for: Existence and significance of anti-HLA-C autoantibodies to primary and persistent platelet transfusion refractoriness in patients with hematologic disorders: a retrospective study from a single centre
Source: Ann Med. 2024 Dec 28;57(1):2446689. doi: 10.1080/07853890.2024.2446689 (PMC11703460; doi:10.1080/07853890.2024.2446689)
Supplement: Supplemental Material [file IANN_A_2446689_SM6790.zip › Suppl_Mat/Supplemental Table 3 .docx]

**Supplemental Table 3. Details of anti-HLA-A, B, C antibodies in patients with positive anti-HLA-C autoantibodies**

|  | Anti-HLA-A antibodies | | | Anti-HLA-B antibodies | | | Anti-HLA-C antibodies | | |
| --- | --- | --- | --- | --- | --- | --- | --- | --- | --- |
|  | High, *N* (gene loci) | Intermediate, N (gene loci) | Low, N (gene loci) | High, *N* (gene loci) | Intermediate, N (gene loci) | Low, N (gene loci) | High, *N* (gene loci) | Intermediate, N (gene loci) | Low, N (gene loci) |
| PT1 | 0 | 2 (69:01,  68:01) | 0 | 7 (35:01,  53:01, 81:01,  51:02, 78:01,  18:01, 51:01) | 28 (48:01, 15:16, 13:01, 44:02, 47:01, 41:01, 58:01, 07:02, 14:01, 44:03, 15:02, 27:05, 37:01, 15:13, 57:03, 27:08, 42:01, 40:06, 45:01, 57:01, 40:01, 52:01, 13:02, 08:01, 49:01, 50:01, 73:01, 40:02) | 8 (14:02,  55:01, 59:01,  38:01, 82:01,  67:01, 15:12,  15:03) | 3 (15:02,  07:02, 06:02) | 5 (18:02,  02:02, 04:01,  17:01, 03:02) | 7 (14:02,  05:01, 08:01,  12:03, 16:01, 03:04, 03:03) |
| PT2 | 0 | 5 (11:01,  11:02, 25:01,  34:01, 01:01) | 19 (36:01, 80:01, 34:02, 24:03, 66:02, 33:01, 33:01, 30:01, 66:01, 24:02, 03:01, 30:02, 31:01, 29:02, 43:01, 74:01, 26:01, 32:01, 29:01) | 6 (15:11, 14:02, 35:01, 82:01, 48:01, 15:02) | 11 (81:01, 78:01, 15:10, 55:01, 46:01, 41:01, 40:01, 45:01, 14:01, 54:01, 18:01) | 18 (50:01, 15:01, 27:08, 42:01, 40:02, 15:03, 73:01, 40:06, 56:01, 07:02, 39:01, 15:12, 08:01, 67:01, 44:02, 57:03, 44:03, 59:01) | 8 (07:02,  14:02, 12:03, 01:02, 16:01, 03:04, 03:02, 08:01) | 1 (03:03) | 7 (17:01,  18:02, 05:01,  06:02, 04:01,  02:02, 15:02) |
| PT3 | 1 (66:02) | 0 | 6 (80:01, 25:01, 32:01, 24:03, 23:01, 24:02) | 25 (07:02, 40:02, 81:01, 27:08, 27:05, 40:06, 40:01,  13:02, 15:12, 48:01, 47:01, 15:11, 15:01, 15:02, 73:01, 13:01, 15:13, 49:01, 67:01,  15:16, 52:01, 46:01, 42:01, 15:10, 15:03) | 8 (55:01, 56:01, 50:01, 57:01, 57:03, 51:02, 54:01, 35:01) | 8 (51:01, 37:01, 8:01, 82:01, 53:01, 58:01, 14:01, 44:02) | 1 (02:02) | 0 | 13 (01:02, 03:03, 14:02, 03:02, 17:01, 03:04, 07:02, 12:03, 18:02, 06:02, 04:01, 16:01, 05:01) |
| PT4 | 15 (36:01, 24:03, 01:01, 24:02, 80:01, 03:01, 31:01, 23:01, 30:01, 11:01, 32:01, 30:02, 33:01, 33:03, 11:02) | 1 (74:01) | 8 (02:06, 68:02, 69:01, 02:01, 43:01, 29:02, 26:01, 29:01) | 28 (49:01, 15:11, 46:01, 13:01, 15:13, 15:16, 57:03, 57:01, 40:06, 15:02, 56:01, 27:08, 41:01, 15:10, 40:01, 52:01, 48:01, 40:02, 15:03, 27:05, 45:01, 13:02, 50:01, 15:12, 44:03, 39:01, 15:01, 14:01) | 6 (58:01, 35:01, 44:02, 51:02, 18:01, 78:01) | 7 (47:01, 53:01, 38:01, 37:01, 07:02, 14:02, 81:01) | 6 (02:02,  15:02, 17:01,  04:01, 06:02,  05:01) | 1 (08:02) | 3 (14:02, 03:04, 03:02) |
| PT5 | 6 (25:01,  11:01, 11:02,  01:01, 80:01,  66:01) | 5 (34:02,  36:01, 26:01,  43:01, 34:01) | 15 (03:01, 66:02, 32:01, 74:01, 31:01, 68:02, 29:02, 33:01, 24:02, 24:03, 29:01, 30:01, 33:03, 30:02, 68:01) | 1 (73:01) | 4 (40:01,  13:02, 48:01,  81:01) | 16 (07:02, 40:02, 40:06, 13:01, 27:08, 27:05, 41:01, 44:02, 45:01, 49:01, 44:03, 15:12, 50:01, 47:01, 15:02,  15:01) | 0 | 0 | 4 (18:02, 07:02, 06:02, 15:02) |
| PT6 | 0 | 0 | 0 | 0 | 0 | 0 | 0 | 0 | 3 (12:03, 07:02, 08:01) |

MFI, mean fluorescence intensity; High: MFI >10000; Intermediate: 5000≤MFI≤10000; Low: 500<MFI<5000
